# Supplementary material for: Seqpare: a novel metric of similarity between genomic interval sets
Source: F1000Res. 2021 Jan 4;9:581. Originally published 2020 Jun 9. [Version 2] doi: 10.12688/f1000research.23390.2 (PMC7808057; doi:10.12688/f1000research.23390.2)
Supplement: Peer review history

# Peer review: Reviewer response for version 2

**DOI:** 10.5256/f1000research.31408.r76397

**Published:** 2021-01-13

**Authors:** Zhaohui Steve Qin

**Affiliations:** 1 Department of Biostatistics and Bioinformatics, Rollins School of Public Health, Emory University, Atlanta, GA, USA

No more questions.

Are the conclusions about the tool and its performance adequately supported by the findings presented in the article?

Partly

Is the rationale for developing the new software tool clearly explained?

Yes

Is the description of the software tool technically sound?

Yes

Are sufficient details of the code, methods and analysis (if applicable) provided to allow replication of the software development and its use by others?

Partly

Is sufficient information provided to allow interpretation of the expected output datasets and any results generated using the tool?

Yes

Reviewer Expertise:

Bioinformatics.

I confirm that I have read this submission and believe that I have an appropriate level of expertise to confirm that it is of an acceptable scientific standard.

# Peer review: Reviewer response for version 1

**DOI:** 10.5256/f1000research.25817.r71030

**Published:** 2020-11-11

**Authors:** Burcak Otlu

**Affiliations:** 1 Department of Cellular and Molecular Medicine and Department of Bioengineering and Moores Cancer Center, University of California, San Diego, La Jolla, CA, USA

The authors have defined a similarity metric for defining similarity between two interval sets. They have justified well why this metric is better than Fisher's exact p-value and odds-ratio. 

 I wonder what is the time complexity of MBM calculation? Does this metric work when there are self-contained intervals (overlapping intervals) in each interval set?  

 Why you choose this metric instead of simply using jaccard index?

 In the text, "6 *ip*s" is written. It would be better to explain "ips" before its usage.

Are the conclusions about the tool and its performance adequately supported by the findings presented in the article?

Yes

Is the rationale for developing the new software tool clearly explained?

Yes

Is the description of the software tool technically sound?

Yes

Are sufficient details of the code, methods and analysis (if applicable) provided to allow replication of the software development and its use by others?

Yes

Is sufficient information provided to allow interpretation of the expected output datasets and any results generated using the tool?

Yes

Reviewer Expertise:

Computational biology, cancer genomics, bioinformatics.

I confirm that I have read this submission and believe that I have an appropriate level of expertise to confirm that it is of an acceptable scientific standard.

# Response

**Published:** 2020-12-10

**Authors:** John Feng

**Affiliations:** Deepstanding LLC, USA

**Comment:** The authors have defined a similarity metric for defining similarity between two interval sets. They have justified well why this metric is better than Fisher's exact p-value and odds-ratio. 

***Response:*** *Thank you for this positive comment.*

**Comment:** I wonder what is the time complexity of MBM calculation? Does this metric work when there are self-contained intervals (overlapping intervals) in each interval set?  

 Why you choose this metric instead of simply using jaccard index?

***Response:*** Thanks for the insightful comments.

***1.*** *Seqpare finds all intersections (total m) by using AIList algorithm with time complexity of O(n log n), where n is the number of intervals in the datasets. The MBM algorithm calculates the similarities of these intersection pairs and then finds the MBM pairs one by one. The time complexity of MBM depends on the cases. If all intersection pairs are simple (Figure 1a), then the complexity is O(m); in the worst case (Figure 1c or Figure 1d), all intersection pairs are correlated and the complexity is*

*O(m* ^*2*^ *). For most real genomic interval sets, the time complexity is close to O(m).*

***2.*** *Seqpare is a general metric of similarity and it works when there are self-contained intervals. Figure 1c and 1d are two cases of self-contained interval sets, where seqpare works well but the p-value based metric breaks down. Also, most of the UCSC interval sets used in this paper contain large number of overlapping intervals.*

***3.*** *Jaccard index is defined as the ratio of Intersection over Union and it takes value in the range of [0, 1], i.e., the Intersection is never larger than the Union and the Union is never zero or negative. Jaccard index can be strictly used for two individual intervals, as explained in the Metric section of the paper. However, it cannot be used directly as similarity metric for two interval sets.*

*For two interval sets, the simple Jaccard metric would be J = N/(N1+N2-N), where N1 and N2 are the number of intervals in the two interval sets, and N is the number of intersections between them. However, the ‘Intersection’ N can be larger than the ‘Union’ (N1+N2-N), and (N1+N2-N) can also be zero or negative: in Figure 1c, N=4, N1=2, N2=2, and (N1+N2-N) is zero; in Figure 1d, N=6, N1=2, N2=3, (N1+N2-N) is -1. Therefore, just like the p-value, simple Jaccard index is not a rigorous similarity metric for interval sets. This also suggests that using p-value or simple Jaccard index to compare interval sets can be misleading and defining a rigorous similarity metric for interval sets is challenging.*

**Comment:** In the text, "6 ips" is written. It would be better to explain "ips" before its usage.

***Response:*** We replaced it as ‘6 intersection pairs’.

# Peer review: Reviewer response for version 1

**DOI:** 10.5256/f1000research.25817.r66632

**Published:** 2020-07-30

**Authors:** Zhaohui Steve Qin

**Affiliations:** 1 Department of Biostatistics and Bioinformatics, Rollins School of Public Health, Emory University, Atlanta, GA, USA

Feng *et al*. described a novel method named seqpare, to measure the similarity between two sets of genomic intervals. I think the authors picked an important problem to work on and they presented a nice solution. The performance, from their studies, seem quite impressive. It is a nice new tool for solving such problems. but I do have a few issues with the paper.

1. I never get what exactly does “self-consistent” really mean. I do not think this is the same as the consistent used in statistics which is an asymptotic property. I think the authors should provide a better explanation of this term and why this is important for such a problem.
2. Genomic intervals, in my opinion, should come with genomic coordinates, to indicate where does it come from in terms of the reference genome. Of course, this will not be the case if the data does not come from a model organism. I am surprised that such information is not used in their method. I think it is a disadvantage if this is not used. Since the problem can be much easier if order is being considered. I am interested to know what do the authors think about this.
3. Figure 1C is not mentioned in the paper. Not sure why.

Are the conclusions about the tool and its performance adequately supported by the findings presented in the article?

Partly

Is the rationale for developing the new software tool clearly explained?

Yes

Is the description of the software tool technically sound?

Yes

Are sufficient details of the code, methods and analysis (if applicable) provided to allow replication of the software development and its use by others?

Partly

Is sufficient information provided to allow interpretation of the expected output datasets and any results generated using the tool?

Yes

Reviewer Expertise:

Bioinformatics.

I confirm that I have read this submission and believe that I have an appropriate level of expertise to confirm that it is of an acceptable scientific standard, however I have significant reservations, as outlined above.

# Response

**Published:** 2020-12-10

**Authors:** John Feng

**Affiliations:** Deepstanding LLC, USA

**Response to Reviewer 1**

**Comment:** Feng et al. described a novel method named seqpare, to measure the similarity between two sets of genomic intervals. I think the authors picked an important problem to work on and they presented a nice solution. The performance, from their studies, seem quite impressive. It is a nice new tool for solving such problems. but I do have a few issues with the paper.

***Response:*** *Thank you for your helpful comments and suggestions. We have revised the paper according to the specific comments, and we have also added a subset of the test data and instructions to the software page to ease the verification of the result.*

**Comment: **I never get what exactly does “self-consistent” really mean. I do not think this is the same as the consistent used in statistics which is an asymptotic property. I think the authors should provide a better explanation of this term and why this is important for such a problem.

***Response:*** *The word "self-consistent" is used here with its literal meaning of "not having parts or aspects which are in conflict or contradiction with each other" (google dictionary). Being self-consistent is necessary for a definition, concept, metric or theory.* *We think it's a useful word and we have explained it in the Introduction. Nevertheless, we did remove it from the title since it may cause confusion without context.*

**Comment:** Genomic intervals, in my opinion, should come with genomic coordinates, to indicate where does it come from in terms of the reference genome. Of course, this will not be the case if the data does not come from a model organism. I am surprised that such information is not used in their method. I think it is a disadvantage if this is not used. Since the problem can be much easier if order is being considered. I am interested to know what do the authors think about this.

***Response:*** *Genomic intervals in the standard BED file format contain essential genomic information such as Chromos* *ome, start coordinate, end coordinate and strand, etc. Like all other tools mentioned in the paper, Seqpare uses BED file format.* *Seqpare does not require the intervals to be sorted, which is an advantage over tools that do require intervals to be sorted.*

**Comment: **Figure 1C is not mentioned in the paper. Not sure why.

***Response:*** *Figure 1 shows four cases and Figure 1c is mentioned as case c in the last paragraph of the Methods section "Seqpare metric". We replaced ‘case c’ with ‘Figure 1c’.*
